# Goat Milk Consumption Enhances Innate and Adaptive Immunities and Alleviates Allergen-Induced Airway Inflammation in Offspring Mice

**DOI:** 10.3389/fimmu.2020.00184

**Published:** 2020-02-18

**Authors:** Hui-Fang Kao, Yu-Chin Wang, Hsiu-Ying Tseng, Lawrence Shih-Hsin Wu, Hui-Ju Tsai, Miao-Hsi Hsieh, Pei-Chi Chen, Wen-Shou Kuo, Li-Fan Liu, Zhi-Gang Liu, Jiu-Yao Wang

**Affiliations:** ^1^Department of Nursing, National Tainan Junior College of Nursing, Tainan, Taiwan; ^2^Center for Allergy and Clinical Immunology Research, College of Medicine, National Cheng Kung University, Tainan, Taiwan; ^3^Graduate Institute of Medical Sciences, China Medical University, Taichung, Taiwan; ^4^Division of Biostatistics and Bioinformatics, Institute of Population Health Sciences, National Health Research Institutes, Zhunan, Taiwan; ^5^Graduate Institute of Medical Sciences, College of Medicine, National Cheng Kung University, Tainan, Taiwan; ^6^School of Chemistry and Materials Science, Nanjing University of Information Science and Technology, Nanjing, China; ^7^Institute of Gerontology, College of Medicine, National Cheng Kung University, Tainan, Taiwan; ^8^Department of Respirology and Allergy, Third Affiliated Hospital of Shengzhen University, Shengzhen, China; ^9^Department of Pediatrics, National Cheng Kung University Hospital, Tainan, Taiwan

**Keywords:** goat milk, immune response, pregnancy, allergic asthma, microbiota

## Abstract

Goat milk (GM), as compared to cow milk (CM), is easier for humans to digest. It also has antioxidant and anti-inflammatory effects and can improve minor digestive disorders and prevent allergic diseases in infants. It is unclear whether GM consumed in pregnant mothers has any protective effects on allergic diseases in infants. In this experimental study with mice, we found GM feeding enhanced immunoglobulin production, antigen-specific (ovalbumin, OVA) immune responses, and phagocytosis activity. The GM-fed mice had an increasing proportion of CD3^+^ T lymphocytes in the spleen. Splenocytes isolated from these animals also showed significantly increased production of cytokines IFN-γ and IL-10. More importantly, GM feeding during pregnancy and lactation periods can confer protective activity onto offspring by alleviating the airway inflammation of allergic asthma induced by mite allergens. There was a remarkably different composition of gut microbiota between offspring of pregnant mice fed with water or with milk (GM or CM). There was a greater proportion of beneficial bacterial species, such as *Akkermansia muciniphila, Bacteroides eggerthii*, and *Parabacteroides goldsteinii* in the gut microbiota of offspring from GM- or CM-fed pregnant mice compared to the offspring of water-fed pregnant mice. These results suggested that improving the nutrition of pregnant mice can promote immunological maturation and colonization of gut microbiota in offspring. This mother-to-child biological action may provide a protective effect on atopy development and alleviate allergen-induced airway inflammation in offspring.

## Introduction

An increasing prevalence of allergic diseases, such as atopic dermatitis, allergic rhinitis, and asthma, as well as food allergies, has been noted in western societies (1, 2). Increasing incidences have also been reported in newly developed Asian countries, such as Taiwan (3, 4). These diseases now affect ~20% of the population worldwide (5, 6); yet the prevalence has increased too rapidly in recent decades to be explained by genetic changes alone (1, 5). This increasing incidence of allergic disease alongside a decreasing incidence of microbial infections in western countries has led to the “hygiene hypothesis” (7). This has been updated to encompass the commensal microbiota in early life (8, 9), which is affected by multiple environmental factors, including the mode of delivery during childbirth (10), breast vs. formula feeding (11), a “Western diet” low in fiber and high in fat content (12), and misuse of antibiotics (13).

Several studies show that children who developed allergies later in life have decreased intestinal microbial diversity, particularly lower levels of *Bifidobacillus* and *Lactobacillus* species in infancy (14). In addition, the pro-inflammatory metabolites produced by dysbiotic microbiota in the neonatal period have been associated with an increasing atopy risk and T-cell differentiation (15). Although breast milk contains numerous allergy-protective bioactive components, such as milk oligosaccharides, polyunsaturated fatty acids, a variety of cytokines of TGF-β and IL-10, and even microbiota (16), there is conflicting evidence on the protective role of breastfeeding in relation to the development of allergic sensitization and allergic diseases (17). A study conducted by Munblit et al. showed that modulation of human breast milk composition may have the potential to prevent allergic disorders in children (18). Human milk composition varies among individuals, which may explain the heterogeneity of these reports. Although, there is evidence that exclusive breastfeeding for 3–4 months reduces the incidence of eczema and is protective against wheezing in the first 2 years of life, there are no short- or long-term advantages for exclusive breastfeeding beyond 3–4 months that have been demonstrated for preventing atopic disease (19).

Previous studies have suggested that goat milk (GM) is easier for humans to digest than cow milk (CM) because its curds are softer (20, 21). The softer curds of GM may be an advantage for adults suffering from gastrointestinal disturbances and ulcers (21). GM contains higher levels of calcium, magnesium, and phosphorous than those of CM and human milk. The higher levels of medium chain triglycerides (MCT) in GM have been recognized as having unique health benefits for infant nutrition (20, 21). Previous studies have demonstrated antioxidant and anti-inflammatory effects of GM (22). For example, Jirillo et al. have shown that GM modulates human peripheral blood mononuclear cells (PBMCs) and polymorphonuclear neutrophils (PMNs) to produce NO, IL-6, IL-10, and TNF-α (22). It is notable that GM is less immunogenic than CM in a murine model of atopy, where the production of IL-4 was lower and IFN-γ was higher from Concanavalin A (ConA)-stimulated splenocytes of GM-fed mice as compared to those of CM-fed mice (23). However, GM is not recommended in CM allergic patients due to the clinically significant cross-allergenicity between CM and GM (24).

Human breast milk contains more than 80 milk oligosaccharides (HMOs). Because of its prebiotic and anti-infective properties, it has been widely recognized as the major source for early life colonization of gut microbiota in infants (25). Recent studies have shown that GM contains the highest level of oligosaccharides among all domestic animals and has significant similarities to human milk oligosaccharides from a structural point of view (26). Though it is clear that a mother's diet influences the health of her fetus in many ways, there is a lack of concrete evidence to link the role of maternal nutrition to the development of allergic diseases in her infants (17, 19). Whether GM consumption by pregnant mothers has atopy protective effects on their newborns is still unclear. This study first evaluates the immune modulation of GM consumption by maternal mice, then it uses pregnant mice and their offspring to verify this hypothesis.

## Materials and Methods

### Animals and Diets

Adult, specific pathogen-free, female BALB/c mice (5–6 weeks old), were purchased from the National Laboratory Animal Breeding and Research Center (Tainan, Taiwan). They were housed in plastic cages with an air filter device and maintained on a standard mouse diet (Lab diet; PMI Feeds, St. Louis, MO, USA) in the Laboratory Animal Center of the College of Medicine, National Cheng Kung University. The composition of the standard diet, which consisted of dry pellets (88%), crude protein (18%), crude fat (3.1%), ash (6.2%), fiber (22%), and carbohydrates (35%). All mice were given *ad libitum* access to deionized water. The GM formula, Mama formulated goat milk (Karihome®), was obtained from Orient EuroPharma Ltd., (Taipei, Taiwan) and manufactured by Dairy Goat Co-operative (NZ) Ltd. (Hamilton, New Zealand). The CM formula was KLIM, powdered milk sold by Nestlé, Switzerland. The GM formula had goat milk protein as the sole protein source, and the CM formula contained cow milk and whey proteins (frequently referred to “whey-enhanced” or “adapted”). In details, the GM formula contained pasteurized goat milk solids (43%), lactose, vegetable oils, minerals, vitamins, acidity regulator (citric acid), choline chloride, L-tryptophan, L-isoleucine, taurine, and L-carnitine. The whey-to-casein ratio was ~20:80, and 60% of the fat was goat milk fat. The CM formula contained cow milk solids (demineralized whey, lactose, skim milk solids, whey solids, and whey protein concentrate), vegetable oils, soy lecithin, minerals, vitamins, acidity regulator (citric acid and/or calcium hydroxide), choline chloride, L-tryptophan, taurine, and L-tyrosine. The whey to casein ratio was ~60:40, and cow fat was not included.

### Experiment and Study Designs

All animal experiments were performed according to protocols approved by the Institutional Animal Care and Use Committee (IACUC No. 105196 and No. 106244). Groups of 12 mice were first used at 6–8 weeks of age. Milk was administered daily to groups of mice by intra-gastric gavage in 200 μL volume. The daily milk intake dose for the mice was calculated from the recommended adult human dose of 25 g/200 mL/60 kg (WHO Dietary recommendations/Nutritional requirements) to 8.5 mg for a 20 g mouse. To evaluate the effect of milk consumption on general immune function, mice were fed with either sterile water (W), GM (low dose 1.6 mg, L; medium dose 8.5 mg, M; and high dose 16.6 mg, H), or CM (8.5 mg; C) for 4 weeks before euthanasia. Mice of control group (N) were fed with normal diet without specific treatment.

To assess the effect of milk consumption on antigen-specific immunological response, groups of mice were fed as described above and were sensitized with an intra-peritoneal (i.p.) injection 50 μg ovalbumin (OVA), 2 μL Complete Freunds Adjuvant (CFA) in 200 μL phosphate-buffered saline (PBS) on day 0, and i.p. [50 μg OVA, 6 μL Incomplete Freunds Adjuvant (IFA) in 200 μL PBS] on day 7. They were then euthanized after 3 weeks. OVA-treated mice were fed with either sterile water (WO), GM (low dose 1.6 mg, LO; medium dose 8.5 mg, MO; and high dose 16.6 mg, HO), or CM (8.5 mg; CO) for 4 weeks before euthanasia. Mice of the control group (N) were fed with normal diet without specific treatment.

**Figure 1:**
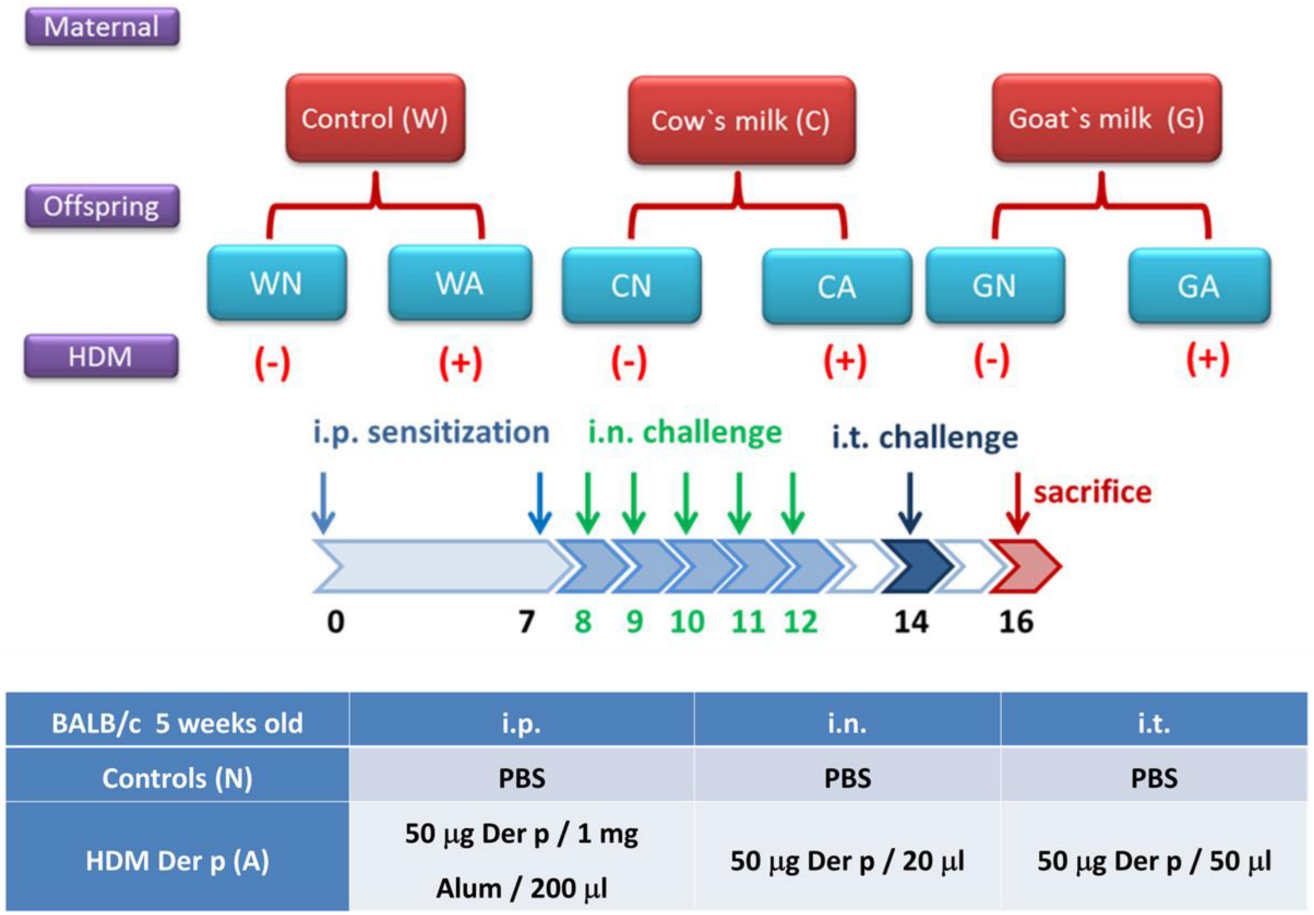
Scheme of study protocol. The classifications of offspring were based on pregnant mother mice fed with water (W), goat milk (G), or cow milk (C) on weaning period (D0) till 2 days after allergen or PBS sensitization and challenge (D16). The offspring mice were divided into two groups: control groups from pregnant mother mice fed with water (WN), goat milk (GN), and cow milk (CN); and HDM-sensitized and challenged groups from pregnant mother mice fed with water (WA), goat milk (GA), and cow milk (CA).

To evaluate the effects of milk consumption by pregnant mice on their offspring, the grouping and mating design was depicted in Figure 1. Female mice were intra-gastrically fed (200 μL) with sterile water (group W), GM (8.5 mg, group G), or CM (8.5 mg, group C) (3 mice/group) after they had been paired with male mice. The total feeding period of female mice began from pairing and continued through pregnancy to the end of a 4-week suckling period. At weaning, the offspring mice were randomly divided into two groups—the control group (WN, GN, and CN) and HDM-stimulating group (WA, GA, and CA)—with 10 mice each. To establish the respiratory allergy model in offspring, they were sensitized with HDM allergen Der p (*Dermatophagoides pteronyssinus*; Allergon, Engelholm, Sweden) on days 0 and 7 by i.p. 200 μL aluminum hydroxide (Al(OH)_3_) [50 μg Der p/mL Al(OH)_3_]. Then, mice were intra-nasally (i.n.) delivered by Der p (50 μg/20 μL PBS) daily (5 days). On day 14, mice were challenged with Der p (50 μg/20 μL PBS) by an intra-tracheal (i.t.) route and were sacrificed 2 days later (Figure 1). Control mice were sensitized with PBS (i.p. and i.n.) and were challenged with PBS (i.t.). On the weaning day, offspring mice were marked W0, C0, and G0 individually.

### Mouse Antibody and Antigen-Specific Antibody Measurements

IgG1, IgG2a, and IgE ELISA kits were purchased from Bethyl Laboratories (Montgomery, TX, USA) and were used according to the manufacturer's recommended protocol. Antigen (OVA)-specific IgA, IgM, IgG, and IgG subclass antibody titers were measured by using an indirect competitive enzyme-linked immunosorbent assay (ELISA) protocol based on previously described methods (27).

### Measurement of Total and Der p-Specific IgE in the Serum

Blood was collected from the cheek facial vein of individual offspring on days 0 and 16. The collected samples were left to stand and clot for 1 h at RT, and they were then centrifuged at 10,000 × g for 30 min to obtain the serum. Serum levels of total and Der p-specific IgE were measured by using an ELISA kit (Mouse IgE ELISA Quantitation Set, E90-115, Bethyl Laboratories, Inc., Montgomery, TX, USA) (28).

### Splenocyte Culture and Cytokine Measurement

A cellular suspension was produced by mincing individual spleens between two sterile glass slides. The red blood cells were lysed with ACK Lysing Solution (Catalog number: A1049201, Thermal Fisher Scientific Inc., Waltham, MA, USA), and the splenocytes were extensively washed and re-suspended in RPMI 1640 containing 10% fetal calf serum, 0.1% penicillin, 0.1% streptomycin, and 0.1% glutamine. Cells (5 × 10^6^ cells/mL) were cultured in 24-well plates at 37°C in 5% CO_2_ and were stimulated with phytohaemagglutinin (1 μg/mL, PHA), ConA (1 μg/mL), or lipopolysaccharide (2 μg/mL, LPS). OVA (10 μg/mL) was used for positive controls and unstimulated cells for background controls. Supernatants were harvested at 48 h and were assayed for the level of IFN-γ, TARC, IL-10, IL-12, and TNF-α concentrations by R&D Systems ELISA (Minneapolis, MN, USA), according to the manufacturer's recommendations. Detection limits were 15 pg/mL for the assays of the abovementioned cytokines.

### Passive Cutaneous Anaphylaxis (PCA)

Specific IgE antibody responses to whey proteins were assessed in triplicate by a PCA test in experimental mice. First, 0.1 ml of twofold dilutions of pooled mouse serum samples was intra-dermally injected into ears of recipient mice. All mice were challenged 48 h later by an intravenous injection of 1 ml of 0.9% saline solution containing 5 mg Evans Blue and 2 mg α-lactalbumin or BSA. The reaction was read 30 min after the challenge. The PCA titer was defined as the highest serum dilution when yielding a positive reaction of at least 5 mm in diameter and expressed as means ± SEM (29).

### Airway Hyperresponsiveness Measurement

To measure mechanical properties of mice airways, mice were injected (i.p.) with 100 mg/kg of pentobarbiturate, and tracheotomies were performed on day 16 (48 h after Der p i.t. challenge). Lung function was determined by using the Scireq Flexivent apparatus (SCIREQ, Scientific Respiratory Equipment Inc., Montreal, Canada). Mice were treated with increasing doses of acetyl-β-methylcholine chloride (0–5 mg/mL) (A2251, Sigma-Aldrich, St. Louis, MO, USA). Methylcholine was aerosolized for ventilation by using an ultrasonic nebulizer for 3 min separately. Respiratory system resistance (Rrs) and elastance (Ers) were calculated by using flexiVent software and fitting the equation with airway resistance (Rn), tissue elasticity (H), and tissue damping (resistance) (G). The data from each treatment group was used to calculate the average response.

### Broncho-Alveolar Lavage Fluid (BALF) and Lung Tissue Examination

The BALF was collected after two times of instillation and aspiration with 1 mL of cold saline into the trachea. BALF was centrifuged at 300 × g for 5 min at 4°C to separate cells and supernatants. The total number of cells in two collections was counted with hemocytometer. Differential cell counts of BALF were performed by cytospin. Cells were stained with Liu's stain solution for microscopic examination, and 200 cells were enumerated. Supernatants were stored at −70°C until assay. To examine the bronchial epithelium inflammation in the lung tissue, lobes were fixed by endotracheal perfusion of alcohol-formalin. After perfusion, the trachea was closed with a suture, and the cardiopulmonary tree was then removed and placed in a 10% neutral buffer formalin (pH 7.4) overnight. Lobes were separated and placed in a cassette for automated paraffin embedding. The paraffin blocks were sectioned into 4–5 μm thickness. Sections were stained with hematoxylin and eosin. Photographs were obtained by a Microscope DP70 (Olympus, Shinjuku, Tokyo, Japan) and DP manager system.

### Analysis of Gut Microbiome Composition by Axiom Microbiome Array

Stool samples were obtained from groups of offspring after the weaning period and HDM allergen sensitization (day 0), and offspring were sacrificed after allergen intra-tracheal challenge for 2 days (Day 16). Stool samples were frozen then stored at −80°C. A QIAamp DNA Stool Mini Kit was used to purify DNA from frozen stool samples according to protocol. DNA quality was evaluated using MaestroNano spectrophotometry (Maestrogen, Las Vegas, NV, USA) in absorbance ratio A260 nm/A280 nm. The Affymetrix GeneTitan® platform was used to identify the diversity of the microbiome with a Thermo Axiom^TM^ Microbiome array, which can detect more than 12,000 species of viruses, bacteria, fungi, protozoa, and archaea (30). Initially, the 200 ng target probes were prepared to detect each DNA sample, which contained at least 20 μL of good-quality DNA (10 ng/μL). These samples were then amplified, fragmented, and hybridized on a chip followed by a single-base extension through DNA ligation and signal amplification. The array was scanned automatically on a GeneTitan Multi-Channel instrument according to manufacturer's instructions (Thermo Fisher, Waltham, MA, USA).

### Microarray Data Analysis

Microarray data were analyzed using MiDAS software (Axiom Microbial Detection Analysis Software), which is based on the Composite Likelihood Maximization Method (CLiMax) algorithm developed at Lawrence Livermore National Laboratory LLNL (31). Probes with signal intensity above the 99th percentile of random control probe intensities and with more than 20% of target-specific probes detected were considered as positives. The microbiome diversity and difference between different samples were calculated by R language. The principal component assay (PCA) was performed by using Python language.

### Statistical Analyses

All analyses were conducted in triplicate. Statistical analysis was performed using GraphPad Prism version 5.0a (GraphPad Software, Inc., La Jolla, CA, USA). Data were analyzed using the Student's *t*-test, Kruskal-Wallis one-way ANOVA, and the Dunn's *post hoc* test. If ANOVA assumptions were violated, the Wilcoxon matched-pairs test would be used. Results are expressed as mean ± SEM. Statistical significance was established at the level of *p* < 0.05.

## Results

### Goat Milk Intake Modulates Immunological Function of Mice

The effects of GM and CM on nutritional immunity were evaluated in mice fed intragastrically with different types of milk for 4 weeks. Control mice were fed with water only. The body weight of the mice increased steadily over the treatment period (weighed once a week) with no difference among groups fed with water, GM (three dosages; L, M, and H), or CM (C) (data not shown). At the end of the treatment period, there was no difference in spleen weight among the six groups (data not shown). Thus, the daily gavage of mice with GM or CM for 4 weeks did not affect weight gain or spleen size.

In contrast, a significant increase in sera immunoglobulin concentration was observed in mice fed with GM or CM. IgA, IgM, and IgG (total) concentrations were significantly higher in cow milk- and goat milk-fed mice compared to control mice (N) (*p* < 0.05) (Table 1A). There was a trend of increased IgG2a levels in mice fed with GM, but this was not statistically significant (Table 1A). Splenocyte proliferation in response to mitogens PHA, Con A, and LPS was without difference between milk-fed groups and control group (Table 1B). Nevertheless, supernatants harvested from 24 h culture of splenocytes in GM treatment groups had increasing concentrations of cytokines (Table 1C). Compared to water and CM groups, GM groups (M and H) had a higher level of IFN-γ after LPS stimulation, a higher level of IL-12 after Con A stimulation, and a higher level of TNF-α after PHA or LPS stimulation, particularly with LPS stimulation (Table 1C). A flow cytometry analysis of spleen cells demonstrated that 4 weeks' milk treatment had limited effect on the proportion of helper T cells (CD3^+^CD4^+^), cytotoxic T cells (CD3^+^CD8^+^), and B cells (CD3^−^CD45R^+^). Although there appeared to be a trend of an increase in B cells, it was not significant (Table 1D). NK-cell activity of splenocytes was increased in mice fed with a low dosage of GM compared to control groups (Table 1E). Phagocytic activity was enhanced in mice fed with GM (all dosages) as compared to control and water-fed groups (Table 1F).

**Table 1:** Immunological functions of mice fed with water, cow milk, and goat milk.

	**Naïve** **(N)**	**Water** **(W)**	**Cow milk** **(C)**	**Goat milk low dose (L)**	**Goat milk medium dose (M)**	**Goat milk high dose (H)**
**(A) IMMUNOGLOBULINS**						
**IgA (μg/mL)**						
Mean ± SEM	128.8 ± 8.6	156.0 ± 11.9	176.3 ± 19.8^*^^a^	207.8 ± 15.5^**^	210.7 ± 23.9^**^^a^	211.4 ± 10.5^**^^a^
**IgM (μg/mL)**						
Mean ± SEM	145.9 ± 14.5	125.1 ± 27.6	271.7 ± 22.2^**^^a^	244.8 ± 24.6^**^^a^	309.2 ± 54.6^**^^a^	28.3 ± 34.0^**^^a^
**Total IgG (μg/mL)**						
Mean ± SEM	27.9 ± 2.9	30.3 ± 4.1	34.7 ± 4.14^*^^a^	38.8 ± 4.1^*^^a^	48.9 ± 7.3^*^^a^	56.4 ± 11.9^*^^a^
**IgG1 (μg/mL)**						
Mean ± SEM	199.8 ± 26.5	167.6 ± 29.1	199.1 ± 28.8	181.9 ± 29.3	165.2 ± 26.1	249.1 ± 27.8
**IgG2a (μg/mL)**						
Mean ± SEM	9.1 ± 1.7	11.3 ± 3.1	15.5 ± 4.7	24.3 ± 10.6	34.4 ± 19.1	30.3 ± 10.1
**(B) SPLEEN CELL PROLIFERATION**						
**At 24 h (ratio)**						
PHA/Medium	1.17 ± 0.08	1.14 ± 0.07	1.21 ± 0.07	1.20 ± 0.09	1.22 ± 0.11	1.20 ± 0.07
Con A/Medium	1.68 ± 0.27	1.32 ± 0.10	1.62 ± 0.31	1.55 ± 0.17	1.61 ± 0.21	1.68 ± 0.22
LPS/Medium	1.09 ± 0.06	1.10 ± 0.02	1.15 ± 0.05	1.12 ± 0.03	1.15 ± 0.05	1.12 ± 0.03
**At 48 h (ratio)**						
PHA/Medium	1.31 ± 0.13	1.27 ± 0.09	1.27 ± 0.07	1.36 ± 0.11	1.40 ± 0.13	1.41 ± 0.10
Con A/Medium	2.33 ± 0.46	2.10 ± 0.31	2.33 ± 0.32	2.92 ± 0.47	2.93 ± 0.51	3.05 ± 0.45
LPS/Medium	1.17 ± 0.08	1.22 ± 0.06	1.23 ± 0.07	1.19 ± 0.03	1.25 ± 0.04	1.24 ± 0.05
**(C) CYTOKINE PRODUCTION**						
**IFN-γ (pg/mL)**						
PHA	117.1 ± 39.6	91.0 ± 22.8	247.2 ± 67.5	227.2 ± 77.4	424.4 ± 157.6	316.0 ± 103.9
Con A	1504 ± 323.8	2121 ± 298.7	1695 ± 284.1	1891 ± 314.0	2192 ± 590.1	1900 ± 331.8
LPS	23.6 ± 5.0	26.9 ± 3.8	25.5 ± 3.5	49.7 ± 14.8	64.8 ± 20.3^*^^a^^,^^*^^b^	76.7 ± 19.0^*^^a^^,^^*^^b^
**IL-12 (pg/mL)**						
PHA	2.88 ± 0.55	2.08 ± 0.67	1.81 ± 0.11	1.85 ± 0.12	1.80 ± 0.19	1.85 ± 0.31
Con A	18.85 ± 6.80	25.68 ± 4.66	27.35 ± 2.40	31.81 ± 5.88	32.33 ± 6.56^*^^a^	31.50 ± 5.93^*^^a^
LPS	2.32 ± 0.37	2.31 ± 0.50	2.04 ± 0.16	1.77 ± 0.14	1.80 ± 0.17	1.55 ± 0.12
**TNF-α (pg/mL)**						
PHA	6.93 ± 1.85	11.27 ± 2.69	10.17 ± 2.67	20.67 ± 8.74	22.76 ± 5.98^*^^a^	21.59 ± 6.26^*^^a^
ConA	172.8 ± 22.47	216.7 ± 16.26	201.7 ± 12.54	224.1 ± 21.37	208.3 ± 22.91	205.1 ± 12.56
LPS	55.17 ± 3.53	6.35 ± 3.90	59.00 ± 4.80	73.48 ± 6.33^*^^a^	79.04 ± 6.72^**^^a^^,^^*^^b^	78.97 ± 4.87^**^^a^^,^^**^^b^
**(D) FLOW CYTOMETRY**						
CD3^+^/CD4^+^	20.40 ± 1.11	21.76 ± 1.74	25.31 ± 2.33	22.80 ± 2.27	19.72 ± 0.45	21.65 ± 2.96
CD3^+^/CD8^+^	10.64 ± 1.46	8.76 ± 1.33	9.95 ± 1.49	9.72 ± 1.11	8.59 ± 1.23	7.90 ± 1.14
CD3^−^/CD45R^+^	30.73 ± 4.88	33.94 ± 3.99	33.91 ± 5.46	36.57 ± 4.18	38.66 ± 3.76	40.75 ± 3.00
**(E) NK CELL ACTIVITY (%)**						
	36.3 ± 7.3	42.2 ± 8.3	42.2 ± 7.7	48.9 ± 8.2^*^^a^	43.8 ± 7.7	39.4 ± 5.8
**(F) PHAGOCYTOSIS (%)**						
	54.8 ± 2.3	56.8 ± 1.8	59.2 ± 2.1	70.7 ± 6.1^*^^a^	69.64 ± 7.2^*^^a^	71.0 ± 14.1^*^^a^

* p < 0.05;

** p < 0.01.

a *as compared to control group*.

b *as compared to cow milk-fed group*.

*Bold values as statistically significance*.

### Goat Milk Intake Increases Antigen-Specific Immunological Response of Mice

The effects of milk consumption on antigen-specific immunological responses were evaluated by extending the above model with OVA immunization protocol. Mice were immunized on Day 14, boosted on Day 21, and sacrificed on Day 28. As described above, there were no significant differences in body weights or spleen size among different treatment groups. A daily milk gavage did not affect food intake compared to the control groups. The immunization protocol induced an antibody response, with the concentrations of total IgM and IgG being increased in sera from all treatment groups compared to non-immunized group (Table 2A). OVA-specific IgA, IgM, IgG, and IgG subclass antibodies also significantly increased in immunized groups, and there were higher levels of OVA-specific IgA and IgG in mice treated with GM compared to non-milk-fed immunized mice. OVA-specific IgA levels were the highest when feeding with medium dosage of GM (Table 2B). After immunization with an OVA antigen, the proliferation activity of splenocytes increased when cultured with PHA, OVA, and LPS in all immunized groups of mice; neither the milk and non-milk-fed groups nor the GM- and CM-fed groups displayed a significant difference in proliferation activity. The LO group, however, showed significantly decreased cell proliferation at 24 h as compared to the non-milk-fed OVA immunized group (O) (Table 2C).

**Table 2:** Antigen-specific immune responses in water, cow milk, and goat milk fed mice.

	**Naïve** **(N)**	**OVA** **(O)**	**OVA with cow milk** **(CO)**	**OVA with low dose goat milk** **(LO)**	**OVA with medium dose goat milk** **(MO)**	**OVA with high dose goat milk** **(HO)**
**(A) TOTAL IMMUNOGLOBULINS**						
**IgA (μg/mL)**						
Mean ± SEM	189.2 ± 19.1	236.1 ± 42.4	169.7 ± 14.1	195.6 ± 24.0	205.2 ± 20.9	204.7 ± 15.3
**IgM (μg/mL)**						
Mean ± SEM	169.6 ± 23.6	455.6 ± 60.7^*^^a^	708.4 ± 51.3^*^^a^^,^^*^^b^	770.7 ± 69.6^**^^a^^,^^*^^b^	798.4 ± 132^**^^a^^,^^*^^b^	768.5 ± 61^**^^a^^,^^*^^b^
**IgG (μg/mL)**						
Mean ± SEM	34.3 ± 10.6	63.4 ± 18.1	99.5 ± 13.8	188.6 ± 35.3^**^^a^^,^^*^^c^	203.0 ± 40.5^**^^a^^,^^*^^c^	166.7 ± 15.2^**^^a^^,^^*^^c^
**(B) OVA-SPECIFIC IMMUNOGLOBULINS (O.D. 450 nm)**						
**Spe IgA**						
Mean ± SEM	0.02 ± 0.01	0.23 ± 0.06	0.28 ± 0.04	0.28 ± 0.04	0.38 ± 0.05^*^^a^	0.26 ± 0.03
**Spe IgM**						
Mean ± SEM	0.04 ± 0.01	0.91 ± 0.19^*^^a^	1.02 ± 0.10^**^^a^	1.20 ± 0.14^**^^a^	1.17 ± 0.22^**^^a^	0.99 ± 0.14^*^^a^
**Spe IgG**						
Mean ± SEM	0.01 ± 0.01	1.76 ± 0.23^*^^a^	2.30 ± 0.06^*^^a^	2.46 ± 0.07^**^^a^	2.46 ± 0.15^*^^a^	2.32 ± 0.12^*^^a^
**Spe IgG1**						
Mean ± SEM	0.01 ± 0.00	2.18 ± 0.23^*^^a^	2.71 ± 0.1^**^^a^	2.60 ± 0.08^*^^a^	2.66 ± 0.15^*^^a^	2.75 ± 0.12^**^^a^
**Spe IgG2a**						
Mean ± SEM	0.01 ± 0.00	0.36 ± 0.10	0.92 ± 0.29^**^^a^^,^^*^^b^	0.59 ± 0.17^*^^a^	0.51 ± 0.11^*^^a^	0.51 ± 0.09^*^^a^
**(C) SPLEEN CELL PROLIFERATION**						
**At 24 h**						
PHA/Medium	1.07 ± 0.04	1.38 ± 0.10	1.22 ± 0.06	1.28 ± 0.09	1.28 ± 0.05	1.27 ± 0.05
OVA/Medium	1.02 ± 0.01	1.21 ± 0.06	1.11 ± 0.04	1.08 ± 0.02^*^^a^	1.10 ± 0.03	1.13 ± 0.02
LPS/Medium	1.04 ± 0.02	1.27 ± 0.09	1.15 ± 0.07	1.14 ± 0.06	1.17 ± 0.07	1.13 ± 0.04
**At 48 h**						
PHA/Medium	1.20 ± 0.08	2.46 ± 0.48	2.13 ± 0.36	1.815 ± 0.31	1.98 ± 0.16	1.83 ± 0.15
OVA/Medium	0.95 ± 0.01	1.30 ± 0.12	1.21 ± 0.08	1.20 ± 0.08	1.17 ± 0.05	1.23 ± 0.05
LPS/Medium	1.15 ± 0.09	1.46 ± 0.21	1.30 ± 0.15	1.18 ± 0.10	1.21 ± 0.12	1.17 ± 0.04
**(D) CYTOKINE PRODUCTION**						
**IFN-γ(pg/mL) 48 h**						
PHA	543.7 ± 156.4	905.1 ± 219.5^*^^a^	838.1 ± 179.3^*^^a^	1214 ± 266.5^*^^a^	1463 ± 255.5^*^^a^	1452 ± 213.2^*^^a^^*^^c^
OVA	4.44 ± 0.41	25.57 ± 3.41^*^^a^	37.52 ± 5.67^*^^a^	78.35 ± 19.57^*^^a^	46.34 ± 7.48^*^^a^	48.47 ± 11.88^*^^a^
LPS	3.81 ± 0.09	15.04 ± 5.93	95.40 ± 31.14^*^^a^	90.87 ± 30.69^*^^a^	223.9 ± 85.95^*^^a^	338.7 ± 143.1^*^^a^
**IL-10 (pg/mL) 48 h**						
PHA	6.68 ± 2.13	17.82 ± 4.16	19.53 ± 3.40	19.13 ± 6.26	23.72 ± 4.11	22.15 ± 2.55
OVA	33.6 ± 8.5	172.3 ± 20.6	239.3 ± 21.1^*^^a^	167.1 ± 45.6	204.2 ± 25.5	305.9 ± 59.5^*^^a^
LPS	3.80 ± 0.13	3.81 ± 0.12	3.99 ± 0.14	3.60 ± 0.13	3.88 ± 0.15	3.80 ± 0.13
**(E) FLOW CYTOMETRY**						
**CD3**^**+**^**(MFI)**	39.62 ± 1.89	36.96 ± 1.42	34.81 ± 1.47	37.70 ± 1.39	40.29 ± 1.64^*^^b^	39.81 ± 1.22^*^^b^
**CD3**^**+**^**/CD4**^**+**^	17.82 ± 1.34	13.37 ± 1.25	14.71 ± 1.24	15.42 ± 1.77	16.70 ± 1.85	16.22 ± 1.62
**CD3**^**+**^**/CD8**^**+**^	14.12 ± 1.20	12.43 ± 1.08	12.19 ± 0.77	12.42 ± 0.57	13.16 ± 0.55	12.94 ± 0.88
**CD3**^**−**^**/CD45R**^**+**^	32.45 ± 2.99	28.64 ± 2.22	29.50 ± 1.34	28.89 ± 1.95	29.65 ± 2.33	29.68 ± 1.68

* p < 0.05;

** p < 0.01.

a *as compared to control group (N)*.

b *as compared to OVA-immunized group (O)*.

c *as compared to cow milk-fed (CO) group*.

*Bold values as statistically significance*.

Splenocytes isolated from OVA-immunized mice produced higher levels of IFN-γ and IL-10 after culturing with PHA and OVA antigen than cells from non-immunized mice. When cells were stimulated with LPS, IL-10 production had no difference between these two groups (Table 2D). Levels of IFN-γ had no significant difference between CM-fed and GM-fed OVA-immunized mice when splenocytes were cultured with OVA and LPS. But there was higher IFN-γ production in high-dose GM-fed mice compared to CM-fed mice as cells cultured with PHA. Splenocytes of mice fed with milk (CM and high-dosage GM) secreted higher levels of IL-10 than those of control mice after stimulating with OVA (Table 2D). After immunization with the OVA antigen, mice fed with GM produced a significantly higher amount of total T cells (CD3^+^) in their spleens, as compared to non-milk- and CM-fed mice (Table 2E). The percentage of other T-cell subpopulations, such as helper T cells(CD3^+^CD4^+^) and cytotoxic T cells (CD3^+^CD8^+^), and B cells (CD3^−^CD45R^+^) in the spleens were not significantly different among the six groups (Table 2E). To assay the recalled antigen immune response, a delayed hypersensitivity reaction for the swelling of mouse ear skin folds was used as described in method section. Supplementary Figure 1A showed that the swelling of the ear skin decreased significantly in GM- and CM-fed mice compared to non-milk-fed mice. A histological examination also showed a significant decrease in epidermis and dermis thicknesses in GM- and CM-fed mice compared to non-milk-fed mice (Supplementary Figure 1B).

### Goat Milk Feeding in Pregnant Mice Confers Protection of HDM-Induced Allergic Airway Inflammation in Offspring

**Figure 2:**
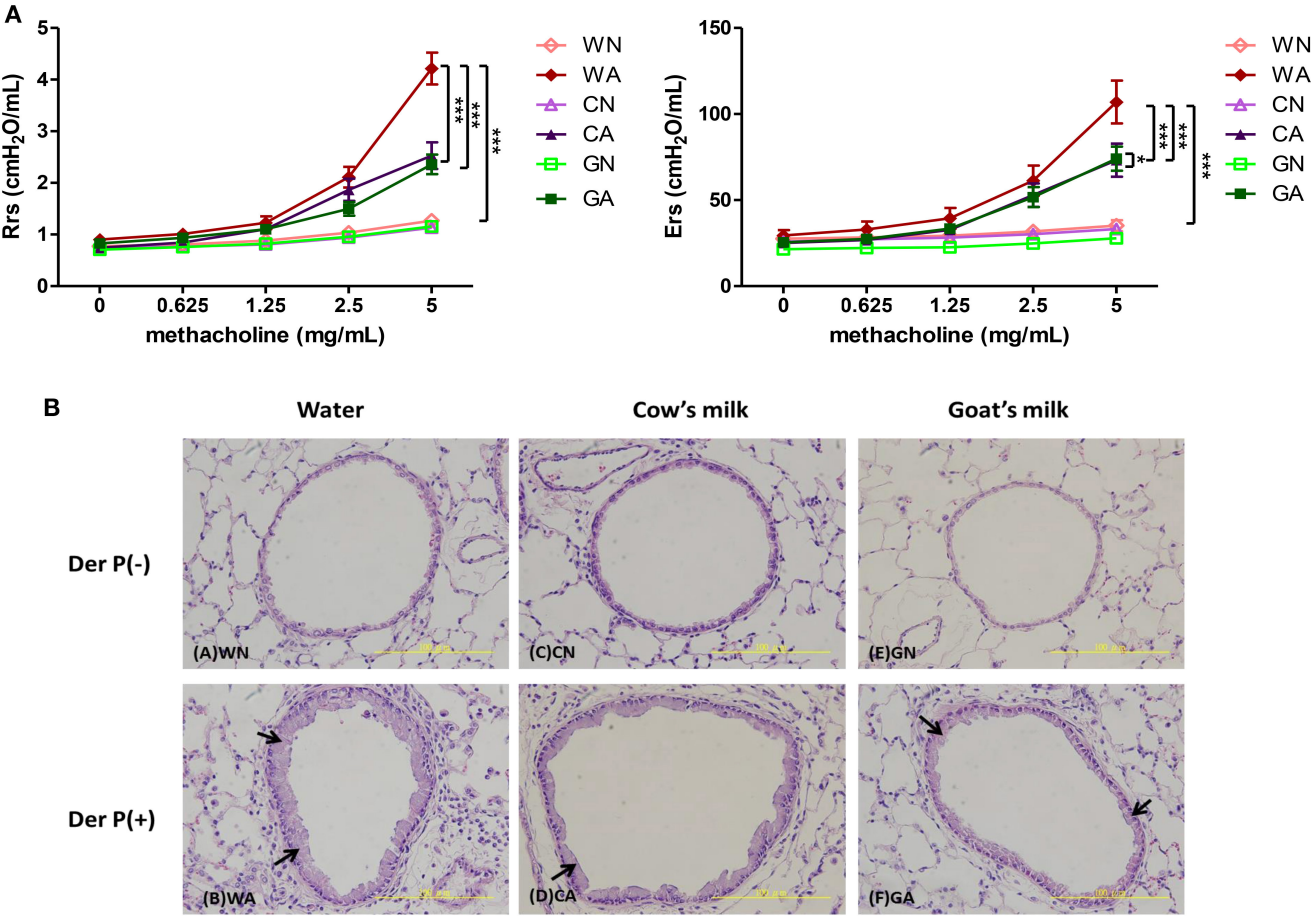
The effects of goat milk feeding in pregnant mother mice on offspring. **(A)** Measurement of airway resistance. **(B)** H&E stains of lung tissues. Each group had 10 mice, and each assay was repeated three times. *P*-value of different groups were compared with those of N groups by Student's *t*-test (***p < 0.001). Pregnant mother mice were fed with sterile water (W), GM (G), or CM (C), and offspring were divided into two groups: control groups from pregnant mother mice fed with water (WN), goat milk (GN), and cow milk (CN); and HDM-sensitized and challenged group from pregnant mother mice fed with water (WA), goat milk (GA), and cow milk (CA).

To explore the protective effect of GM- or CM-fed pregnant mice on allergen-induced airway inflammation in their offspring, we administrated the maternal group with water, CM, or GM daily from mating until offspring were weaned at 4 weeks of age. Offspring mice were divided into six groups (female and male, *n* = 6 in each group) according to the maternal mice feeding models. Sensitized (i.p.), intra-nasal (i.n.), and intra-tracheal (i.t.) challenges with HDM (Der p) or with PBS were carried out on the offspring (Figure 1). There was no difference in body weight among the groups of offspring throughout the study (data not shown). The HDM-treated groups (WA, CA, and GA) with exposure to methylcholine induced significantly increasing airway resistance at day 14. However, airway resistance was less severe in GA and CA groups (GA: Rrs, 2.358 cm H_2_O/mL and Ers, 62.26 cm H_2_O/mL, CA: Rrs, 2.527 cm H_2_O/mL and Ers, 85.45 cm H_2_O/mL) throughout pregnancy and lactation. The decrease in resistance was significant at the concentrations of 2.5 and 5 mg/ml methylcholine inhalation as compared to that of WA group (Rrs, 4.213 cm H_2_O/mL and Ers, 137.4 cm H_2_O/mL, *p* < 0.05) (Figure 2A). In a lung histological examination, non-HDM-sensitized mice (WN, CN, and GN) had minimal inflammatory cell infiltration and lower mucosal thickness (arrow) in the bronchial epithelium than those of HDM-sensitized mice. After being challenged with HDM, GA and CA groups showed significantly decreased inflammatory cell infiltration (12 ± 5 and 7 ± 3 cell/HPF, respectively) and mucosa thickness as compared to those of WA group (35 ± 7 cells/HPF) (Figure 2B).

**Figure 3:**
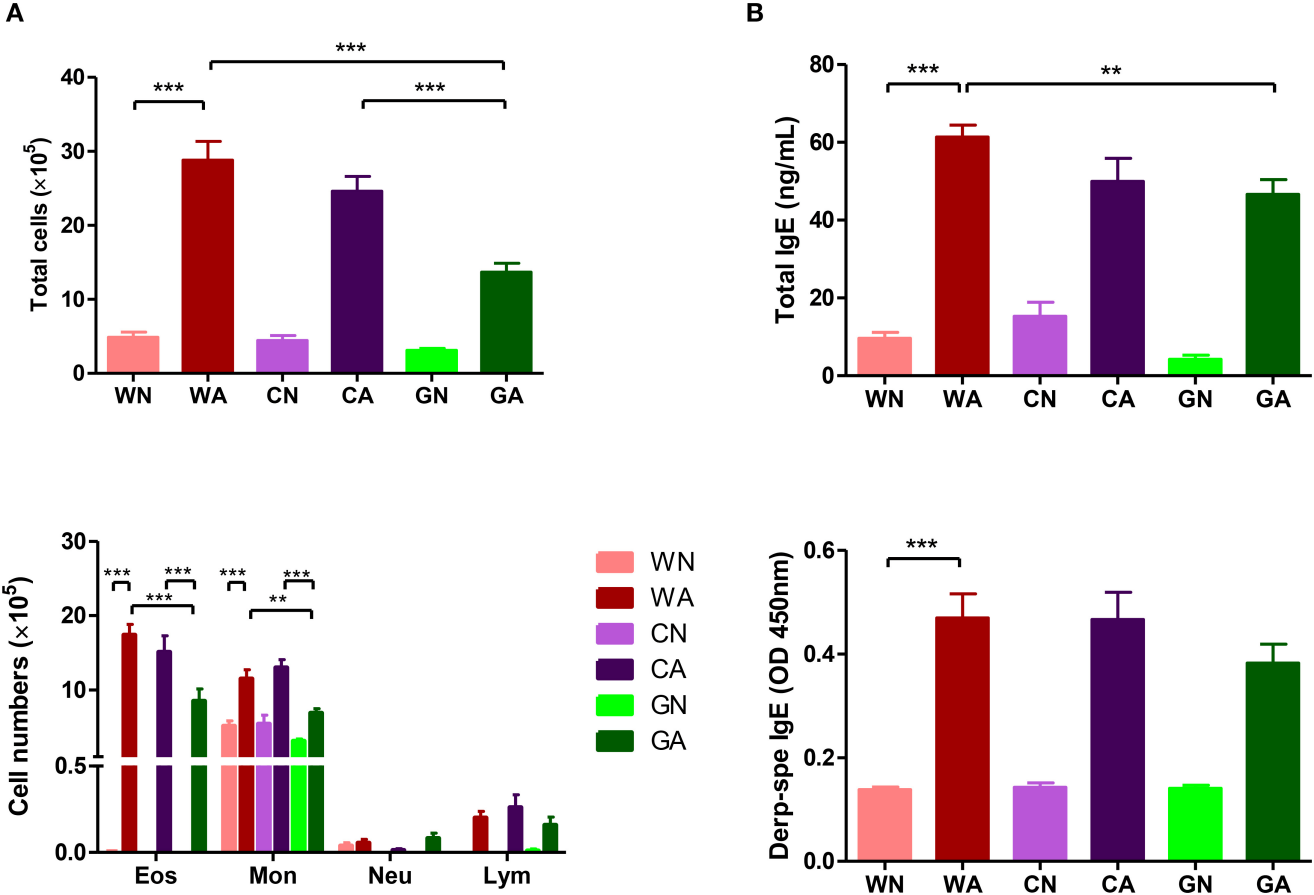
HDM allergen-induced lung inflammation and sera IgE levels in offspring. **(A)** Total infiltrated cells and the number of eosinophils in BALF **(B)** total IgE and Der p-specific IgE levels in sera. Each group had 10 mice, and each assay was repeated three times. *P*-value of different groups were compared with those of N groups by Student's *t*-test (**p < 0.01 and ***p < 0.001).

**Figure 4:**
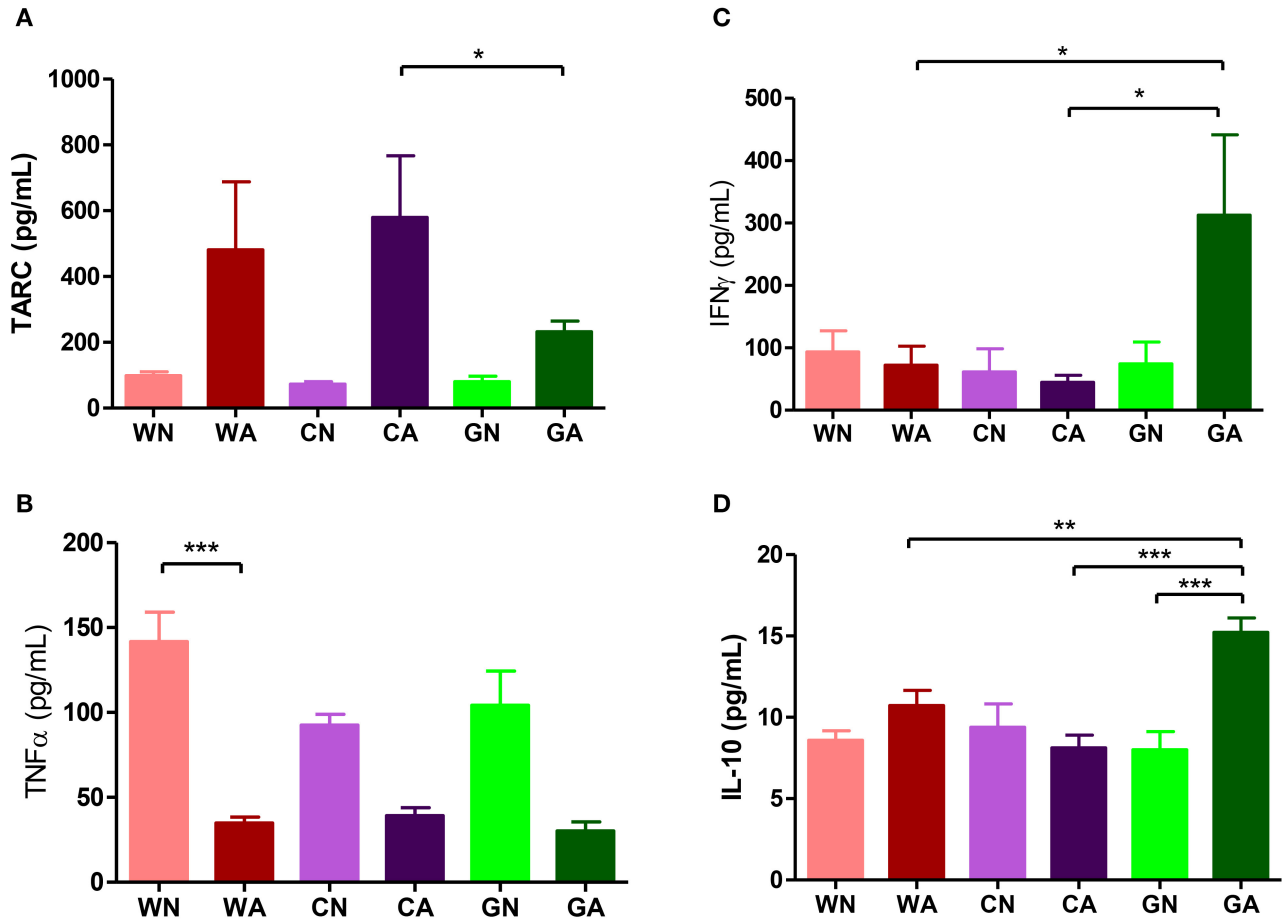
Cytokines production in offspring. **(A,B)** Cytokines levels (TARC and TNF-α) of BALF. **(C,D)** Cytokines production (INF-γ and IL-10) of culture supernatants from PHA-stimulated splenocytes. Each group had 10 mice, and each assay repeated for three times. *P*-value of different groups were compared with those of N groups by Student's *t*-test (*p < 0.05; **p < 0.01; and ***p < 0.001).

Further analysis of BALF from HDM-sensitized mice showed that there were increasing numbers of eosinophils, monocytes, and lymphocytes. This confirmed the inflammatory cell infiltration into the lungs. However, BALF from the GA group had lower total cell infiltration levels and fewer numbers of eosinophils compared to those of WA and CA groups (Figure 3A). In mice primed with respiratory allergen (HDM), there were significantly higher levels of total IgE and HDM-specific IgE antibodies than those of non-sensitized mice (Figure 3B). However, the GA group had significantly lower levels of total IgE compared to WA group (*p* < 0.05). There was a trend of lower levels of Der p-specific IgE antibodies in the GA and CA groups (Figure 3B). Assays of cytokine production in BALF showed lower levels of TARC in the GA group compared to the WA and CA groups (Figure 4A). The levels of TNF-α in BALF were more reduced in HDM-sensitized mice compared to non-HDM-sensitized mice (Figure 4B). There was no significant difference in TNF-α among HDM sensitized and challenged mice. Splenocytes collected from GA group produced the highest levels of IFN-γ following PHA stimulation among the six groups (Figure 4C). Furthermore, splenocytes from GA mice produced significantly higher levels of IL-10 after PHA stimulation as compared to cells from the WA and CA groups of mice (*p* < 0.05; Figure 4D).

### Goat Milk Feeding Induces Gut Microbiota Change in HDM-Sensitized and Challenged Offspring

**Figure 5:**
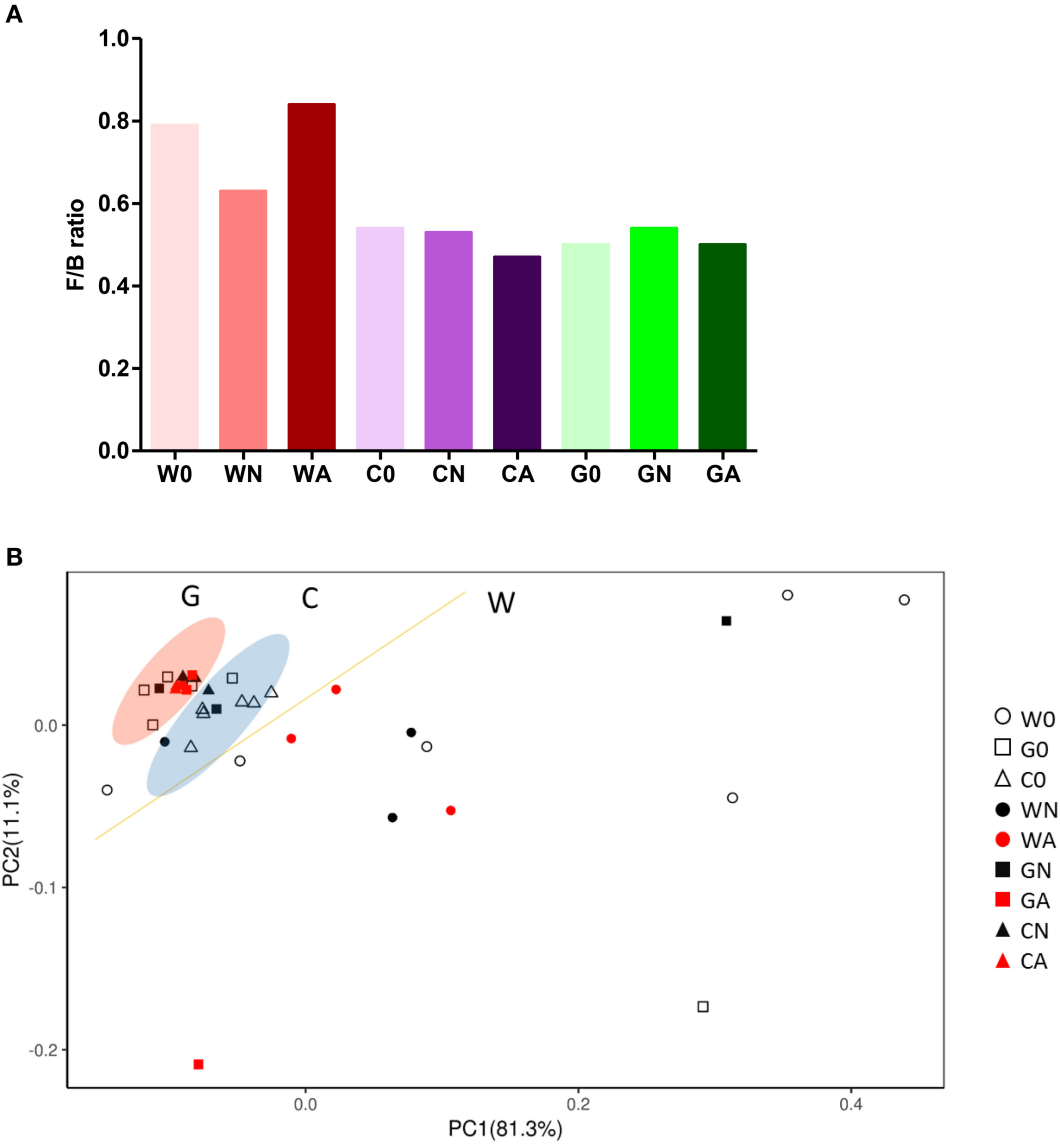
Gut microbiota change in groups of offspring with or without HDM-sensitization and challenge. **(A)** The ratio of *Firmicutes* to *Bacteroidetes* (F/B ratio) in gut microbiota **(B)** Bi-plot representing the weighted Principal Coordinates Analysis (PCoA), pair-wise UniFrac distances showing clustering of bacterial groups from stool samples in groups of offspring.

**Figure 6:**
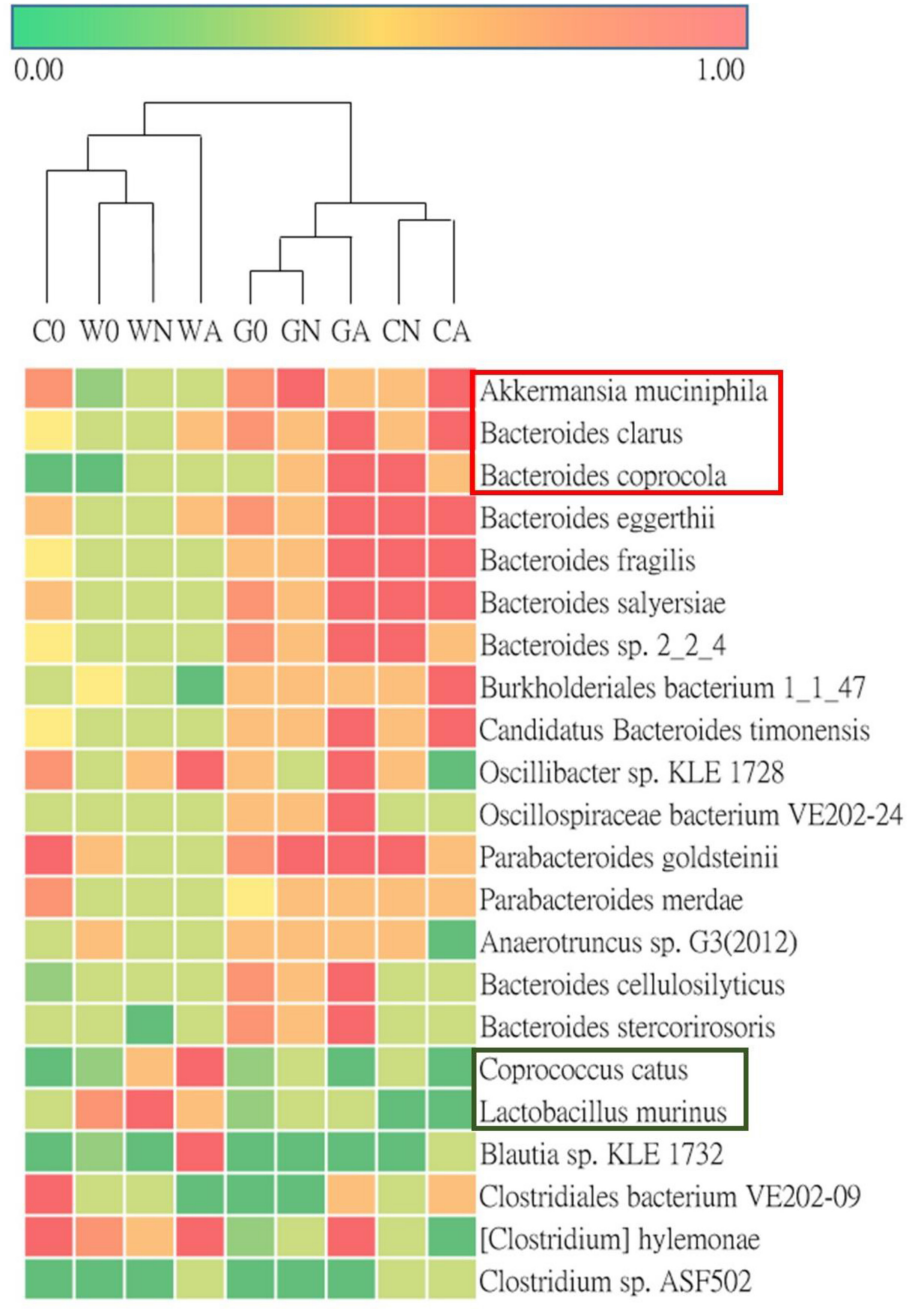
Heatmap for the ratio of detected bacterial strains of microbiota in groups of offspring.

To analyze gut microbiota among groups of weaned offspring and the effect of gut microbiota on allergen-induced airway inflammation, we collected the stools of the offspring before allergen sensitization (day 0) and 2 days after i.t. allergen challenge (day 16). The detection of the cDNA of stools using Applied Biosystems™ Axiom™ Microbiome Array found the class of *Bacteroidia, Clostridia, Flavobacteriia, Bacilli, Deferribacteres, Verrucomicrobiae*, and *Gammaproteobacteria* as well as some unclassified viruses (Table 3). Comparing the ratio of phyla *Firmicutes* to *Bacteroidetes* (F/B ratio), the water-fed (W0) group had a higher F/B ratio (0.79) than the GM-fed (G0) (0.50) and CM-fed (C0) groups (0.54) at Day 0. After HDM allergen sensitization and challenge there was a remarkable increase in the F/B ratio in water-fed mice (0.63 in WN vs. 0.84 in WA), while there was no change of F/B ratio in GM-fed (GN vs. GA) and CM-fed mice (CN vs. CA) (Figure 5A). A Weighted Principal Coordinates Analysis (PCoA) for the microbiome of each sample based upon the UniFrac method was performed to compare the overall composition of the bacterial community within the samples (Figure 5B). Gut microbiota of offspring from water-fed mice had a wider spread in PCoA, while offspring from GM- or CM-fed mice, though not overlapping, clustered in the upper left corner of PCoA, suggesting that these gut microbiotas were more abundant and relating to each other. It was also notable that there was no significant change in the abundance and β-diversity in the gut microbiota between non-sensitized and Der p allergen sensitized/challenged offspring from GM- or CM-fed mice, while gut microbiota of offspring from water-fed mice showed greater change in PCoA between WN and WA. The results from heatmap plots showed there were more dominant strains in the gut microbiota of offspring from GM- and CM-fed mice but less in the offspring of water-fed mice (Figure 6). Examples of dominant bacterial strains include *Akkermansia muciniphila, Bacteroides eggerthii*, and *Parabacteroides goldsteinii*, which had been reported to be beneficial to human health. In contrast, *Coprococcus catus, Lactobacillus murinus, Blautia sp. KLE 1732*, and *Clostridiales bacterium VE202-09* were found to be dominant in the gut microbiota of offspring from water-fed mice but less in the offspring of GM- or CM-fed mice (Supplementary Figure 2).

**Table 3:** Goat milk feeding in perinatal period induces gut microbiota change in HDM-sensitization and challenged offspring.

**Superkingdom**	**Phylum**	**Class**	**D0W**	**D0G**	**D0C**	**D16WN**	**D16WN**	**D16GN**	**D16GA**	**D16CN**	**D16CA**
Bacteria	Bacteroidetes	Bacteroidia	45	47	45	45	44	46	45	46	46
Bacteria	Firmicutes	Clostridia	30	25	23	24	33	24	24	26	23
Bacteria	Bacteroidetes	Flavobacteriia	3	3	3	3	1	2	3	3	3
Bacteria	Firmicutes	Bacilli	8	0	3	6	5	2	0	0	0
Bacteria	Deferribacteres	Deferribacteres	1	1	1	1	1	1	1	1	1
Bacteria	Verrucomicrobia	Verrucomicrobiae	1	1	1	1	1	1	1	1	1
Bacteria	Proteobacteria	Gammaproteobacteria	0	1	1	0	0	0	0	0	0
Viruses	Unclassified	Unclassified	1	0	1	0	0	0	1	0	0

## Discussion

Bioactive compounds presenting in food are called nutraceuticals or functional foods. They are beneficial to the human body in many aspects and may go beyond their nutritional roles. Goat milk contains several bioactive compounds that might be useful in relieving cardiovascular disease, metabolic disorders, neurological degeneration, and promoting the establishment of intestinal microbiotas (32). In host immunity, when pathogens invade human body, B cells will generate antibodies to target specific antigens (33). Casein phosphopeptides of GM can increase the level of IgA in stool, which suggests a positive effect on mucosal immunity. Lactoferrin in GM has been demonstrated to play an important role in increasing the activity of NK cells and increasing the phagocytic activity of phagocytes (34). GM can also trigger IL-10, TNF-α, and IL-6 production in blood cells (35).

Our results showed that GM-fed mice could enhance the immune response in antibody production (IgA, IgM, and IgG subclasses) and phagocytosis activity promotion. Compared to CM-fed mice, there were more IFN-γ, IL-12, and TNF-α cytokine production in the culture supernatant of stimulated splenocytes in GM-fed mice. When mice were immunized with a specific antigen (OVA), GM-fed mice, but not CM-fed mice, had more antigen-specific antibodies (IgA, IgM, IgG, and IgG subclasses) than water-fed mice. There was a significant increase in IFN-γ and IL-10 production in the culture supernatant of stimulated splenocytes as well as an increase in the amount of CD3^+^ T lymphocytes in GM-fed mice. More importantly, we found these enhancements of the immune response in innate and adaptive immunities in pregnant mice; mice fed with GM in particular could pass immunity to their offspring to alleviate allergen-induced airway inflammation of allergic asthma. These offspring from pregnant mice fed with GM or CM showed a drastic change of gut microbiota composition after weaning, compared to offspring of water-fed mice. We suspected that GM feeding during pregnancy and lactation might change the composition of breast milk and confer immunological maturation and colonization of gut microbiota on offspring, and this might suppress atopy development and downregulate airway inflammation.

Relationships among a wide spectrum of bioactive factors, such as proteins, polyunsaturated fatty acids, oligosaccharides, microbial content, metabolites, and micronutrients present in breast milk and allergy development in infants have attracted more attention (36–39). Various maternal exposures during pregnancy, such as immunization, dietary patterns, vitamin D, omega-3 fatty acids, and/or probiotics may affect breast milk composition and thereby influence the early colonization of gut microbiota and infant health (16, 40). Early microbial colonization is essential to infants' metabolic and immunological development (41). There is a direct link between microbial colonization and the risk of non-communicable diseases in later life, including allergies (42). After birth, the transfer of microbiota continues during lactation, and it is considered as the cause of differences in gut microbiota between exclusively breast-fed and formula-fed infants during the first month of life (43). In clinical trials, oral administration of bacterial strains to lactating mothers modulated both human milk composition and infant's gut microbiota. For instance, intake of *Lactobacillus reuteri* led to its detection in the mother's milk and infant stool (44). Another study found that giving *Lactobacillus rhamnosus* to mothers during pregnancy and lactation can reduce the risk of allergy development (45). Probiotic intake during pregnancy and lactation also induced specific changes in infant *Bifidobacterium* colonization and affected breast milk microbiota composition, oligosaccharides, and lactoferrin (46).

While it is clear that mother's diet influences the health of her fetus, there is currently no concrete evidence in the role of maternal nutrition and the development of allergic diseases in children. As compared to formula feeding, there is clear evidence that breastfeeding can increase gut microbial biodiversity in infants. Whether GM consumption during pregnancy and lactation can induce changes of intestinal microbiota in newborns has never been explored. One clinical study (47) was conducted to compare the composition of the stool microbiotas of infants (<2 years old) fed with GM formula, CM-based formula, or breast milk. The results of the beta-diversity analysis showed that gut microbiotas and *Lachnospiraceae* populations were more similar between breast/goat milk comparisons than those between breast/cow milk comparisons. This similarity appeared to be based on the predominance of *Ruminococcus gnavus* among *Lachnospiraceae* in breast/goat milk-fed microbiotas. Our study showed there were significant differences in the intestinal microbiota compositions (PCoA analysis) and decreased *Firmicutes*/*Bacteroidetes* (F/B) ratio in the offspring of GM- or CM-fed pregnant mice compared to those offspring of water-fed mice. Besides, allergen sensitization and challenge induced slight changes in the composition of gut microbiota and F/B ratio in offspring of milk-fed mice, in contrast to the wide swings of change in the offspring of water-fed mice. These results were consistent with previous research that the resilient characteristics and atopy-protective role of colonized gut microbiota could confer from milk-fed maternal mice to their offspring during pregnancy and lactation periods (48).

The abundance of bacterial species, such as *A. muciniphila* and *P. goldsteinii*, in the offspring's gut microbiota of GM- or CM-fed mice had multiple regulatory functions on glucose metabolism in diabetes and obesity as well as anti-inflammatory action in inflammatory bowel diseases (49–51). *Bacteroides eggerthii* and *Bacteroides fragilis* were reported to be associated with propionate production in human intestine (52). Propionate is a short-chain fatty acid and is suggested to be associated with IL-10-producing regulatory T (Treg)-cell differentiation in gut-associated lymphoid tissues (53). Recently, it had been found that there were reduced *A. muciniphila* and *Faecalibacterium prausnitzii* levels in the intestinal microbiota of children with allergic asthma (54), which might explain the anti-asthma protective role of GM-fed offspring with increasing levels of *A. muciniphila* in their gut microbiota.

In conclusion, this study showed that GM consumption could enhance immune function and antigen-specific immune response in mice. Furthermore, maternal GM consumption during pregnancy and lactation periods could affect the composition of gut microbiota in offspring and protected them against atopy and allergen-induced airway inflammation (Supplementary Figure 3). We believe these findings have important clinical implications in the improvement the nutrition of pregnant mothers and components of their breastmilk. Future trials are needed to prove this concept in order to promote maternal health and perinatal nutrition and to reduce allergic diseases in infants.

## Data Availability Statement

The microarray data has been uploaded to the GEO—GSE144086. Other raw data supporting the conclusions of this article will be made available by the authors, without undue reservation, to any qualified researcher.

## Ethics Statement

The animal study was reviewed and approved by the Institutional Animal Care and Use Committee (IACUC No. 105196, and No. 106244), College of Medicine, National Cheng Kung University.

## Author Contributions

H-FK, Y-CW, and H-YT conducted the experiments of the immunological studies. H-FK, M-HH, and P-CC conducted the experiment of the mouse model of allergic asthma. LW, L-FL, and H-JT for microbiota assays, bio-information, and statistics analysis. W-SK and Z-GL for technical advice. H-FK and J-YW for experimental design and writing up manuscript.

### Conflict of Interest

The authors declare that the research was conducted in the absence of any commercial or financial relationships that could be construed as a potential conflict of interest.

## Supplementary Material

Supplementary Figure 1 **(A)** Thickness of skin, epidermis, and epidermis of ears and **(B)** H&E stain of ear skins after passive cutaneous anaphylaxis (PCA) test in groups of mice. Each group had 12 mice and each assay was repeated three times. *P*-value of different groups were compared with those of N groups by Student's t-test (^*^*p* < 0.05; ^**^*p* < 0.01; ^***^*p* < 0.001).

Supplementary Figure 2 The ratio of representing bacterial strains in gut microbiota in different groups of offspring, with or without HDM-sensitization & challenge. Female mice were fed with sterile water (W), GM (G), or CM (C) and offspring were divided into two groups: control group (WN, GN, CN) & HDM-stimulating group (WA, GA, and CA). On weaning day, offspring were marked W0, C0, and G0 individually.

Supplementary Figure 3 Graphic summary of goat milk effects on immune responses and allergy diseases in offspring.
