# Supplementary figures and images for: Goat Milk Consumption Enhances Innate and Adaptive Immunities and Alleviates Allergen-Induced Airway Inflammation in Offspring Mice
Source: Front Immunol. 2020 Feb 18;11:184. doi: 10.3389/fimmu.2020.00184 (PMC7040033; doi:10.3389/fimmu.2020.00184)

# Supplementary Fig. 1

(A)

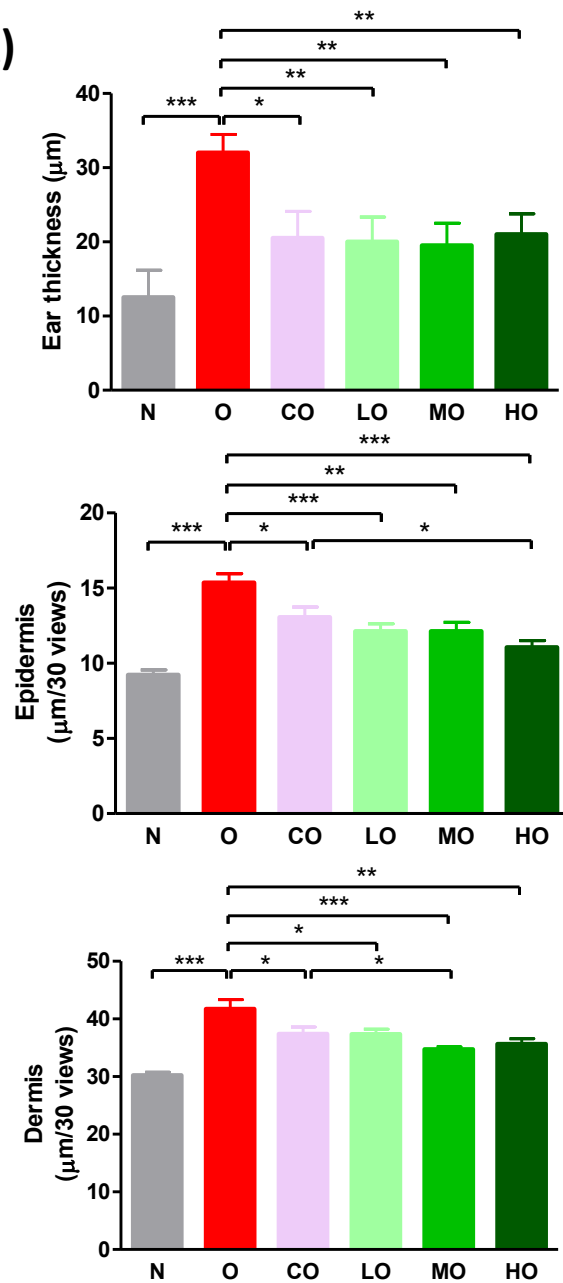

(B)

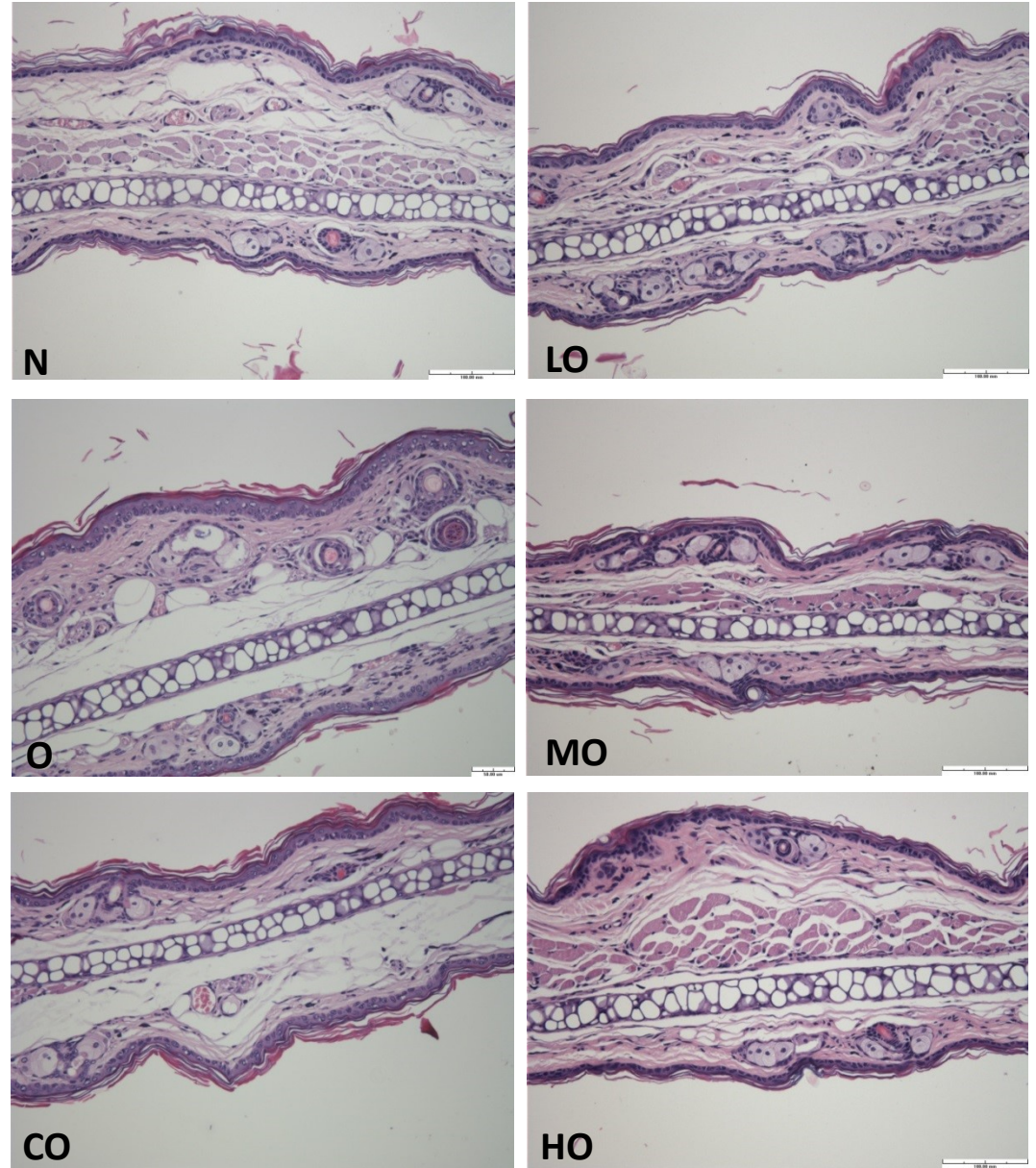

Supplement: Supplementary Figure 1 — (A) Thickness of skin, epidermis, and epidermis of ears and (B) H&E stain of ear skins after passive cutaneous anaphylaxis (PCA) test in groups of mice. Each group had 12 mice and each assay was repeated three times. P-value of different groups were compared with those of N groups by Student's t-test (*p < 0.05; **p < 0.01; ***p < 0.001). [file Image_1.pdf]

Supplementary Fig. 2

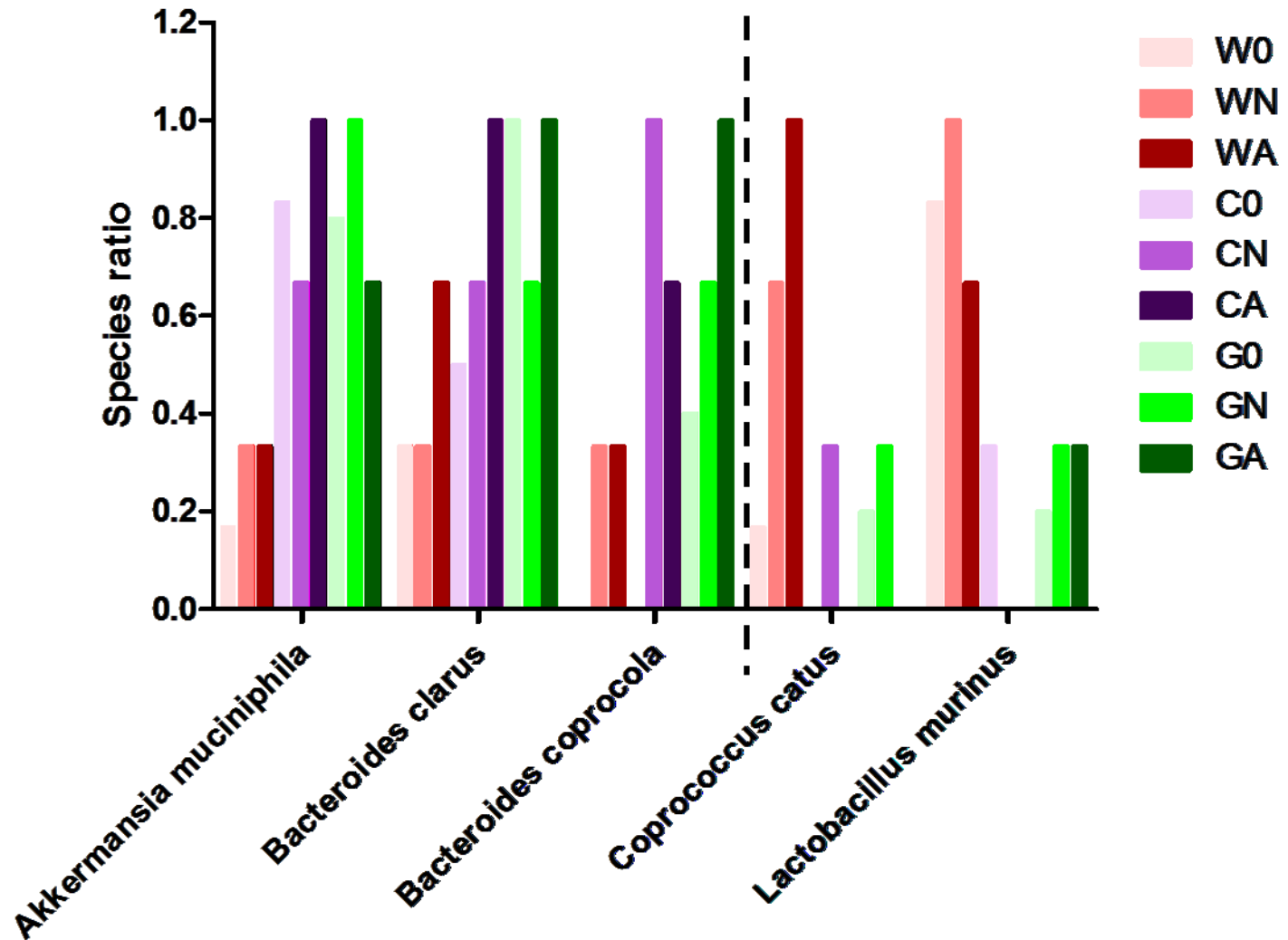

Supplement: Supplementary Figure 2 — The ratio of representing bacterial strains in gut microbiota in different groups of offspring, with or without HDM-sensitization & challenge. Female mice were fed with sterile water (W), GM (G), or CM (C) and offspring were divided into two groups: control group (WN, GN, CN) & HDM-stimulating group (WA, GA, and CA). On weaning day, offspring were marked W0, C0, and G0 individually. [file Image_2.pdf]

## Supplementary Fig. 3

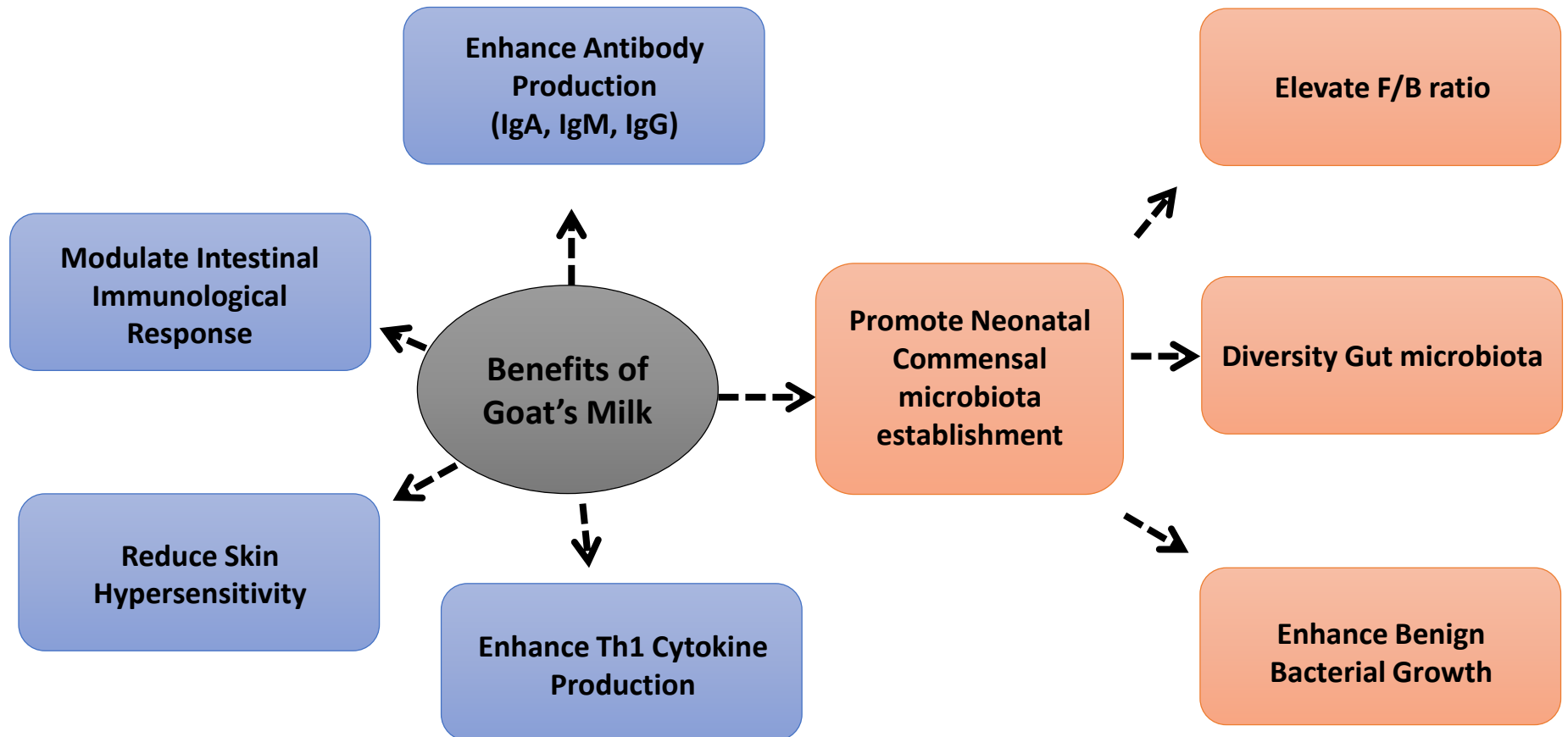

Supplement: Supplementary Figure 3 — Graphic summary of goat milk effects on immune responses and allergy diseases in offspring. [file Image_3.pdf]
